# Intraoperative hypotension and postoperative delirium among older high-risk patients undergoing major noncardiac surgery: a retrospective single-centre cohort study

**DOI:** 10.1016/j.bjao.2025.100500

**Published:** 2025-10-15

**Authors:** Inaame Ettoumi, Daniel Pearce, Theodora Wingert, Amelie Delaporte, Brenton Alexander, Ravi Pal, Jason Tang, Nancy M. Boulos, Yann Gricourt, Janice Boktor, Maziar M. Nourian, Tristan Grogan, Cecila Canales, Dan Cole, Robert A. Whittington, Maxime Cannesson, Alexandre Joosten

**Affiliations:** 1 Department of Anesthesiology, Université Paris-Saclay, Hôpital Bicetre, Assistance Publique Hôpitaux de Paris, Le Kremlin Bicêtre, France; 2 Department of Anesthesiology & Perioperative Medicine, David Geffen School of Medicine, University of California Los Angeles, Los Angeles, CA, USA; 3 David Geffen School of Medicine, University of California Los Angeles, San Diego, CA, USA; 4 Department of Anesthesiology, University of California San Diego, La Jolla, CA, USA; 5 Department of Medicine Statistics Core, David Geffen School of Medicine, University of California Los Angeles, Los Angeles, CA, USA

**Keywords:** arterial pressure, blood pressure, confusion, outcome, postoperative

## Abstract

**Background:**

Intraoperative hypotension has been associated with postoperative complications, but its relationship with postoperative delirium remains debated.

**Methods:**

This single-centre retrospective cohort study included adults (≥60 yr) with ASA physical status score of 3 or 4 undergoing major noncardiac surgery, with documented Confusion Assessment Method assessments. Patients with a history of neurosurgery, stroke, dementia, or neurocognitive disorders were excluded. The primary exposure was the cumulative duration of a mean arterial pressure <65 mm Hg (minutes). The primary outcome was postoperative delirium within 7 days, diagnosed via Confusion Assessment Method. Multivariable logistic regression was used to assess the association between intraoperative hypotension and delirium, adjusting for confounders.

**Results:**

Among 5171 patients included from 2013–2024, 632 (11.8%) developed delirium. The median (Q1–Q3) duration of surgery and time with mean arterial pressure <65 mm Hg were 281 (199–430) min and 28 (9–61) min, respectively. In models adjusted for patient characteristics and perioperative factors, intraoperative hypotension was associated with increased odds of delirium (odds ratio per 60 min, 1.12; 95% confidence interval, 1.01–1.24; *P*=0.038). However, after adjusting for year of surgery, the association was attenuated and no longer statistically significant (odds ratio, 1.06; 95% confidence interval, 0.95–1.18; *P*=0.320). Both intraoperative hypotension exposure and delirium incidence declined significantly over the study period.

**Conclusions:**

Although intraoperative hypotension initially appeared to be associated with postoperative delirium, this association was no longer significant when accounting for temporal improvements in perioperative care. Intraoperative hypotension may represent a marker of historical practice patterns rather than an independent causal driver of delirium.

Delirium is an acute syndrome characterised by fluctuating disturbances in attention, awareness, and cognition. It commonly occurs within 24–72 hours after surgery, particularly among older patients.^1^ Incidence rates vary widely from 10% to 50% depending on the type of surgery and patient population.^2^, ^3^, ^4^ It leads to increased perioperative morbidity and mortality, prolonged hospital stays, and higher healthcare costs, placing a significant burden on both the patients and the healthcare system.^5^, ^6^, ^7^

Although multifactorial in origin, growing evidence implicates intraoperative hypotension (IOH) as a potentially modifiable contributor to delirium.^8^, ^9^, ^10^ IOH, usually defined as a mean arterial pressure (MAP) below 65 mm Hg, has been strongly associated to end-organ dysfunction, including acute kidney and myocardial injury.^11^ Importantly, IOH may also result in cerebral hypoperfusion, thereby contributing to the development of postoperative delirium.^12^ Under normal conditions, cerebral autoregulation maintains stable cerebral blood flow across a wide range of blood pressures.^13^ However, this mechanism becomes impaired with ageing and in the presence of comorbidities such as hypertension, hypercholesterolemia, and diabetes (any types). These factors reduce vascular compliance and compromise autoregulatory capacity, making the brain more susceptible to hypoperfusion during hypotensive episodes.^13^ Furthermore, IOH may lead to cerebral hypoxia, disruption of the blood–brain barrier, and neuroinflammation—all implicated in the pathogenesis of delirium.^4^ Repeated or prolonged hypotensive events may also exacerbate preexisting neurodegenerative conditions, further increasing vulnerability in at-risk individuals.

Despite these proposed mechanisms, relatively few published studies have specifically examined the association between IOH and postoperative delirium in patients undergoing major noncardiac surgery and existing results have been inconsistent.^8^, ^9^, ^10^^,^^14^, ^15^, ^16^, ^17^ Consequently, the extent to which IOH contributes to delirium remains uncertain.

To address this gap, we conducted a retrospectively analysis of our institutional database focusing on high-risk older patients with ASA physical status score of 3 or 4. We hypothesised that within this vulnerable population, IOH would be significantly associated with increased risk of developing postoperative delirium.

## Methods

### Design and setting

This single-centre, retrospective, cohort study was approved by the University of California, Los Angeles (UCLA) Institutional Review Board on 1 April 2024 (IRB no. 24-000493) with a waiver of informed consent. Deidentified data were obtained from consecutive patients who underwent major noncardiac surgery at Ronald Reagan UCLA Medical Center between 1 January 2013 and 1 April 2024. Data collection was sourced from electronic health records (EHRs) at the UCLA Perioperative Data Warehouse.^18^ This study adheres to the Strengthening the Reporting of Observational Studies in Epidemiology (STROBE) guidelines and the checklist is given in Supplementary material 1.

### Inclusion and exclusion criteria

All consecutive unique patients older than 60 yr who were undergoing noncardiac surgery under general anaesthesia and had an ASA score of 3 or 4 (defined as high-risk) were identified in the UCLA database from January 2013 to April 2024. To focus on an older, high-risk surgical population, we restricted inclusion to ASA physical status 3–4. Patients recorded as ASA 2 or 5 at the preoperative assessment (or final day-of-surgery update, when available) were excluded per protocol. ASA classification was abstracted from the clinician-completed preoperative anaesthesia record; when multiple entries existed, the final pre-induction ASA assignment was used. Only patients with records of delirium screening, using the Confusion Assessment Method (CAM), were included in the analysis. Patients with a history of dementia, cognitive impairment, stroke, and transient ischaemic attack (TIA) and those who underwent a neurosurgical procedure were not included, to be consistent with existing literature. Preexisting diagnoses were ascertained from three concordant sources: (1) the structured preoperative anaesthesia evaluation template (clinician-completed comorbidity checklist), (2) the EHR problem list, and (3) International Classification of Diseases, 10th revision (ICD-10) encounter/billing diagnoses from the preoperative period. A condition was considered present if documented in any source by a clinician.

### Anaesthesia protocol

Intraoperative monitoring was standardised according to institutional guidelines. All patients received standard ASA monitoring, including oxygen saturations, noninvasive blood pressure (NIBP), five-lead electrocardiogram, and end-tidal CO_2_ monitoring. Depth of anaesthesia monitoring and invasive blood pressure monitoring were left to the discretion of the attending anaesthesiologist. Intraoperative blood pressure management was not protocolised; anaesthesia teams titrated fluids and vasopressors according to routine clinical judgement, patient comorbidities, and surgical stimuli. Consistent with contemporary evidence linking IOH to adverse outcomes,^11^^,^^19^, ^20^, ^21^ a MAP ≥65 mm Hg was routinely targeted in the past 5–6 yr.

### Exposure

The primary exposure was IOH, quantified as the cumulative duration of a MAP <65 mm Hg, expressed in minutes. The duration of MAP <65 mm Hg in minutes was calculated for each patient. We selected 65 mm Hg *a priori* as the primary IOH threshold because it is widely used in perioperative studies of hypotension and adverse outcomes.^21^^,^^22^ For regression modelling, the duration was scaled to 60-min (1-h) increments to improve clinical interpretability and because modelling duration per minute resulted in an odds ratio (OR) very close to 1.00, making the effect difficult to meaningfully detect.

### Confounding variables

Potential confounders were selected *a priori* based on clinical relevance and previous literature on risk factors for postoperative delirium. Patient characteristics included age (years), sex, and BMI (kg m^−2^). Surgical risk was represented by ASA physical status (ASA 4 *vs* lower) and type of surgery (thoracic, orthopaedic, abdominal, head and neck, with vascular surgery as the reference). Comorbidities included chronic kidney disease, anaemia, arterial hypertension, diabetes (any type), alcohol use, depression, coronary artery disease, and atrial fibrillation, defined using preoperative diagnoses, ICD-10 codes, and/or relevant laboratory or imaging criteria as available. Supplementary material 2 shows the definition used for the confounding factors.

Intraoperative variables included use of an arterial line, cumulative duration of anaesthesia (hours), intraoperative midazolam use, opioid administration (yes/no), ketamine use (yes/no), total fluid administered (millilitres), estimated blood loss (millilitres), fluid balance (millilitres), total phenylephrine dose (micrograms), and total norepinephrine dose (milligrams). Because both IOH and postoperative delirium incidence declined over the study period, year of surgery was additionally included in the model to account for potential temporal confounding. Preoperative and intraoperative exposures were entered into the multivariable regression model simultaneously with the primary exposure (cumulative minutes with MAP <65 mm Hg) to adjust for potential confounding effects.

### Outcomes

The primary outcome was postoperative delirium, defined as a single positive CAM score in the first 7 days after surgery. CAM assessments were performed by registered bedside nurses who receive formal institutional training. Per UCLA protocol (routine care), CAM is assessed at least once every 12-h shift (i.e. twice daily) in all patients ≥60 yr old during the postoperative period until patient discharge. The CAM screening tool is embedded within the EHR and includes structured prompts for attention, orientation, and fluctuation in mental status. Only patients with at least one documented CAM assessment were included in the study. A single positive CAM was considered sufficient for classification as postoperative delirium, in line with previous cohort studies and guidelines recognising the fluctuating nature of the condition. Patients without a documented CAM were not included in the IRB-approved data extract, and a complete, linkable denominator of unscreened patients with comparable baseline covariates was not available across all locations.

### Data collection

Deidentified data were sourced from the perioperative patient data warehouse and electronic medical records of all included patients. UCLA Health’s Discovery Data Repository has systematically collected and stored deidentified medical records since 2013 which reflects the starting date for our data collection.

All intraoperative MAP values were extracted from the UCLA Perioperative Data Warehouse, which integrates data from the electronic anaesthesia record system across all operating rooms.

Blood pressure was measured either via intermittent NIBP monitoring using an upper arm cuff or continuously via an invasive arterial line, as determined by the clinical team in charge of the patients. To ensure data uniformity, all MAPs, regardless of the source, were interpolated to 1-min intervals. If a value was not recorded at a given minute, the last known MAP was carried forward until a new value was recorded (‘last observation carried forward’ method), following conventions used in previous large-scale perioperative studies. Artifacts were excluded using predefined thresholds:

• MAP values <30 or >200 mm Hg were considered physiologically implausible.
• Sudden changes of ≥80 mm Hg within a 1-min interval were flagged and excluded as likely artifacts.
• Non-numeric or null entries were excluded.

The intraoperative period was defined from ‘anaesthesia start’ to ‘anaesthesia end’ timestamps recorded by the anaesthesia provider. The duration spent below a given MAP threshold (e.g. <65 mm Hg) was computed as the cumulative number of interpolated 1-min epochs below that threshold. This standardised and automated processing pipeline was validated and used in several previous retrospective studies at our institution and minimises missingness and bias caused by manual charting errors.

Alternative definitions of IOH were explored and different MAP thresholds, including:

• % MAP <65 mm Hg (the percentage of case time patients spent below the 65 mm Hg threshold)
• The duration of MAP <70 mm Hg (in minutes)
• % MAP <70 mm Hg (the percentage of case time patients spent below the 70 mm Hg threshold)

### Statistical analysis

Continuous variables were summarised as medians with interquartile ranges (Q1–Q3), and categorical variables as counts with percentages. We presented descriptive statistics based on four groups: quartile 1–4 of MAP <65 mm Hg expressed in minutes duration. Duration of MAP <65 mm Hg was categorised into quartiles to facilitate descriptive comparisons across exposure levels and to identify potential dose-response trends without assuming linearity.

Baseline and perioperative characteristics were compared across these groups using *t*-tests, one-way analysis of variance (anova) for continuous variables, and χ^2^ tests for categorical variables; when any expected cell count was <5, Fisher’s exact test (with simulated *P*-values) was used.

Multivariable logistic regression models were used to assess the association between IOH and postoperative delirium, adjusting for prespecified patient characteristics, comorbidity, and intraoperative confounders and year of surgery (see ‘Confounding variables’ subsection).

We prespecified adjustment covariates using a directed acyclic graph to avoid over-adjustment. Variables occurring *post-exposure* that plausibly lie on the pathway between IOH and delirium (e.g. postoperative hypoxaemia, infection, uncontrolled pain, excess opioid exposure, dehydration, sleep disruption, and reduced mobilisation) were **not** included in the primary multivariable model to preserve estimation of the *total* effect of IOH. Where relevant perioperative factors were incompletely captured in structured fields (e.g. anticholinergic administration, benzodiazepines, sensory impairment), we did not include them as covariates in the primary analysis. Results from multivariable models are presented as adjusted ORs with corresponding 95% confidence intervals (CIs) and *P*-values.

All statistical analyses were conducted using IBM SPSS Statistics for Windows, Version 29.0 (IBM Corp., Armonk, NY, USA). A *P*-value of <0.05 was considered statistically significant.

## Results

### Study cohort

**Fig 1:**
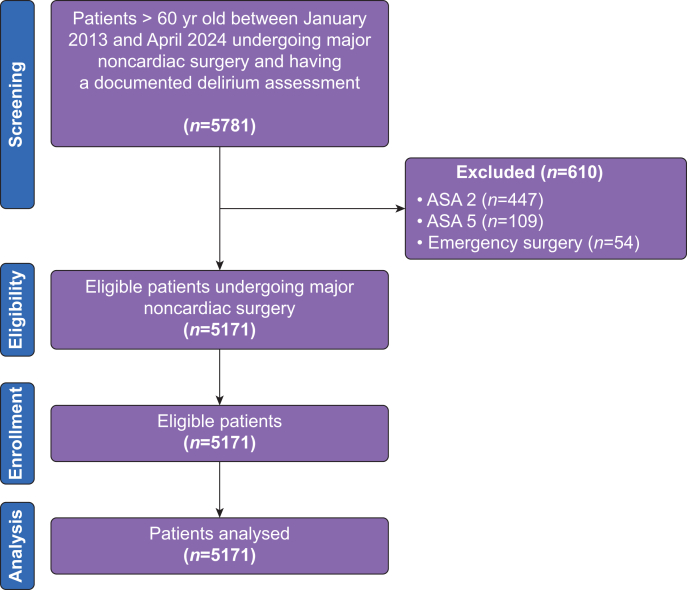
Flow chart.

A total of 5171 unique patients aged ≥60 yr with an ASA physical status classification of 3 or 4 who underwent major noncardiac surgery between 1 January 2013 and 1 April 2024 and had documented postoperative CAM screening were included in the study. A flow diagram outlining the study selection process is presented in Figure 1. Per the prespecified eligibility criteria, ASA physical status 1–2 and 5 were not included. Patients with ASA physical status 1–2 were excluded to focus on higher-risk patients, whereas patients with ASA physical status 5 often remained intubated after surgery, rendering CAM screening unavailable or unreliable.

Among the included patients, 66% had an ASA physical status classification of 3, whereas 34% had a score of 4. The majority were male (58%). Intraoperatively, approximately 54% of patients underwent arterial line blood pressure monitoring. Baseline characteristics in the fourth quartile of cumulative MAP <65 mm Hg were generally comparable with those in the other quartiles; however, a slightly higher cardiovascular risk profile was observed in this group, with an increased proportion of patients having a history of myocardial ischaemia. Table 1, Table 2 reflect the baseline and intraoperative characteristics according to duration in minutes of MAP <65 mm Hg quartiles. The median (Q1–Q3) duration of surgery was 281 (199–430) min.

**Table 1:** Patients’ baseline characteristics according to a MAP <65 mm Hg quartiles. Data are presented as *n* (%) or median (interquartile range) as appropriate. CABG, coronary artery bypass graft; MAP, mean arterial pressure. ∗Surgeries were classified into six categories: (1) thoracic (all types of thoracic procedures); (2) orthopaedic (primarily hip and knee arthroplasty and spine surgery); (3) abdominal (hepatic/pancreatic, urological, and gynaecological procedures); (4) head and neck (predominantly ear, nose, and throat tumour resection with reconstruction); (5) vascular (mainly aortobifemoral bypass and abdominal aortic aneurysm repair, open or endovascular); and (6) other (for plastic-bronchoscopy-Cath lab procedures and all other types of high-risk surgery).

Variables	Overall (*n*= 5171)	Duration of time with MAPs < 65 (minutes)				*P*-value
		Quartile 1 (0–9)	Quartile 2 (10–28)	Quartile 3 (29–61)	Quartile 4 (62–523)	
		(*n*=1305)	(*n*=1298)	(*n*=1293)	(*n*=1275)	
Age (years)	71 (65–77)	70 (65–77)	71 (65–77)	71 (65–77)	71 (65–77)	0.749
Height (inches)	67 (63–70)	67 (64–70)	67 (63–70)	66 (63–69)	67 (64–70)	<0.001
Weight (kg)	75 (63–88)	77 (63–89)	75 (64–89)	75 (62–88)	74 (63, 86)	0.004
Body mass index (kg m^−2^)	25.9 (22.8–29.9)	25.9 (22.5–30.0)	26.2 (23.0–30.2)	26.2 (22.8–29.9)	25.4 (22.7–29.5)	0.021
Female sex	2145 (42%)	498 (38%)	539 (42%)	578 (45%)	530 (42%)	0.009
ASA physical status 3	3411 (66%)	897 (69%)	888 (68%)	824 (64%)	802 (63%)	0.001
Preoperative creatinine (mg dL^−1^)	0.9 (0.7–1.3)	1.0 (0.7–1.3)	0.9 (0.7–1.3)	1.0 (0.7–1.4)	1.0 (0.7–1.4)	0.013
Type of surgery∗						<0.001
• Thoracic	1360 (26.3%)	317 (24.3%)	390 (30.0%)	344 (26.6%)	309 (24.2%)	
• Orthopaedics	486 (9.4%)	118 (9.0%)	125 (9.6%)	136 (10.5%)	107 (8.4%)	
• Abdominal surgery	1411 (27.3%)	407 (31.2%)	340 (26.2%)	360 (27.8%)	304 (23.8%)	
• Head and neck	1142 (22.1%)	261 (20.0%)	246 (19.0%)	271 (21.0%)	364 (28.5%)	
• Vascular surgery	641 (12.4%)	171 (13.1%)	169 (13.0%)	150 (11.6%)	151 (11.8%)	
• Other	131 (2.5%)	31 (2.4%)	28 (2.2%)	32 (2.5%)	40 (3.1%)	
Chronic kidney disease	959 (19%)	235 (19%)	196 (16%)	263 (21%)	265 (22%)	<0.001
Preoperative Haemoglobin (g dL^−1^)	11.5 (9.5–13.2)	11.8 (9.8–13.5)	11.8 (9.7–13.4)	11.2 (9.2–13.0)	11.1 (9.3–12.9)	<0.001
Anaemia	3029 (64.7%)	709 (61%)	692 (59%)	814 (69%)	814 (70%)	<0.001
Arterial hypertension	2332 (45.1%)	725 (56%)	622 (48%)	536 (42%)	449 (35%)	<0.001
Diabetes (any types)	269 (5.2%)	36 (2.8%)	59 (4.5%)	70 (5.4%)	104 (8.2%)	<0.001
Alcohol use	63 (1.2%)	7 (0.5%)	13 (1.0%)	20 (1.5%)	23 (1.8%)	0.016
Depression	202 (3.9%)	34 (2.6%)	41 (3.2%)	65 (5.0%)	62 (4.9%)	0.002
CABG surgery	59 (1.1%)	12 (0.9%)	13 (1.0%)	16 (1.2%)	18 (1.4%)	0.631
Ischaemic cardiomyopathy	484 (9.4%)	87 (6.7%)	128 (9.9%)	115 (8.9%)	154 (12.1%)	<0.001
Atrial fibrillation	103 (2.0%)	33 (2.6%)	23 (1.8%)	26 (2.1%)	21 (1.7%)	0.371
Preoperative midazolam	1524 (30%)	329 (25%)	362 (28%)	378 (29%)	455 (36%)	<0.001

**Table 2:** Patients’ perioperative characteristics according to a MAP <65 mm Hg quartiles. Data are presented as *n* (%) or median (interquartile range) as appropriate. MAP, mean arterial pressure. ∗Any patients who received either fentanyl remifentanil or hydromorphone during surgery. ^†^ Acute kidney injury is defined using the The Kidney Disease: Improving Global Outcomes (KDIGO) criteria.

Variables	Overall (*n*=5171)	Duration of time with MAPs < 65 (minutes)				*P*-value
		Quartile 1 (0–9)	Quartile 2 (10–28)	Quartile 3 (29–61)	Quartile 4 (62–523)	
		(*n*=1305)	(*n*=1298)	(*n*=1293)	(*n*=1275)	
Anesthesia duration (min)	281(199–430)	223 (165–301)	256 (190–360)	295 (208–437)	421 (283–596)	<0.001
Arterial line insertion	2813 (54)	468 (36)	663 (51)	796 (62)	886 (70)	<0.001
MAP <65 mm Hg (min)	28 (9–61)	3 (0–6)	18 (14–23)	42 (35–51)	100 (77–146)	<0.001
MAP <65 mm Hg (%)	9 (3–20)	1 (0–3)	7 (5–10)	14 (10–20)	27 (19–40)	<0.001
MAP <70 mm Hg (min)	61 (26–120)	11 (4–20)	42 (31–56)	84 (66–106)	178 (134–249)	<0.001
MAP <70 mm Hg (%)	21 (10–37)	5 (2–9)	16 (11–22)	29 (21–38)	47 (36–60)	<0.001
Number of patients receiving opioid∗	4522 (87)	1130 (87)	1149 (89)	1129 (87)	1114 (87)	<0.001
Total intraoperative fluids (ml)	1000 (1000–1700)	1000 (1000–1000)	1000 (1000–1340)	1000 (1000–1900)	1500 (1000–2982)	<0.001
Estimated blood loss (ml)	100 (100–200)	100 (100–100)	100 (100–150)	100 (100–260)	100 (100–500)	<0.001
Total phenylephrine during surgery (mcg)	800 (100–4132)	200 (0–1750)	775 (100–3559)	1000 (200–4810)	1489 (300–6984)	0.001
Total norepinephrine during surgery (mcg)	0.0 (0.0–0.0)	0 (0–0)	0 (0–0)	0 (0–0)	0 (0–419)	<0.001
Number of patients receiving ketamine	1032 (20.0)	201 (15)	276 (21)	257 (20)	298 (23)	<0.001
Incidence of acute kidney injury^†^	1642 (31.8)	320 (25)	343 (26)	460 (36)	519 (41)	<0.001
Hospital length of stay (days)	10 (5–19)	8 (3–16)	9 (4–17)	11 (6–20)	12 (7–23)	<0.001

### Primary analysis

Delirium occurred in 632 patients (11.8%). The median (Q1–Q3) duration of MAP <65 mm Hg was 28 (9–61) min. The incidence decreased over the 11-yr study period, paralleling a decline in median IOH duration (Supplementary material 3). In models adjusted for patient characteristics and perioperative factors, longer cumulative IOH duration was associated with higher odds of postoperative delirium (OR per 60 min, 1.12; 95% CI, 1.01–1.24; *P*=0.038). However, after additionally adjusting for year of surgery to account for temporal trends, the association was attenuated and no longer statistically significant (adjusted OR per 60 min, 1.06; 95% CI, 0.95–1.18; *P*=0.320). Independent predictors of delirium included increasing age, male sex, ASA 4 physical status classification, chronic kidney disease, preoperative anaemia, type of surgery, and year of surgery.

Table 3 displays the multivariable adjusted Odd Ratios associated with postoperative delirium.

**Table 3:** Multivariable adjusted Odds Ratios for postoperative delirium (year included). There are 207 missing values for chronic kidney disease, 492 missing values for anaemia, and 113 missing values for atrial fibrillation. 95% CI, 95% confidence interval; MAP, mean arterial pressure; OR, odds ratio.

Variables	Postoperative delirium	
	OR (95% CI)	*P*-value
MAP < 65 mm Hg (minutes)	1.01 (0.99–1.03)	0.320
Preoperative midazolam	0.74 (0.59–0.94)	0.012
Use of an arterial line	0.89 (0.72–1.09)	0.247
Duration of anaesthesia	1.00 (1.00–1.00)	0.273
Age (years)	1.03 (1.02–1.04)	0.000
Body mass index (kg m^−2^)	0.98 (0.96–1.00)	0.022
Male sex	1.22 (1.01–1.49)	0.040
ASA physical status 4	1.77 (1.45–2.17)	0.000
Type of surgery		<0.001
Other *vs* vascular	3.29 (1.83–5.91)	0.000
Thoracic *vs* vascular	2.25 (1.58–3.19)	0.000
Orthopaedic *vs* vascular	2.26 (1.51–3.37)	0.000
Abdominal *vs* vascular	1.41 (0.99–1.99)	0.055
Head and neck *vs* vascular	1.08 (0.75–1.57)	0.671
Chronic kidney disease	1.41 (1.12–1.77)	0.003
Anaemia	1.42 (1.14–1.77)	0.002
Arterial hypertension	0.95 (0.78–1.15)	0.593
Diabetes (any type)	0.78 (0.49–1.23)	0.284
Alcohol use	0.57 (0.15–2.26)	0.427
Drug abuse	3.47 (1.03–11.65)	0.044
Depression	1.04 (0.64–1.69)	0.868
Coronary artery disease	1.45 (0.69–3.05)	0.322
Atrial fibrillation	1.45 (0.83–2.51)	0.191
Use of intraoperative opioids	0.82 (0.63–1.06)	0.132
Total fluid in (ml)	1.00 (1.00–1.00)	0.152
Blood loss (ml)	1.00 (1.00–1.00)	0.040
Fluid balance (ml)	1.00 (1.00–1.00)	0.212
Total phenyephrine (mcg)	1.08 (0.86–1.36)	0.518
Total norepinephrine (mg)	1.36 (1.07–1.74)	0.013
Use of intraoperative ketamine	0.84 (0.65–1.09)	0.191
Year of surgery	0.91 (0.88–0.95)	0.000

## Discussion

In this large, 11-year single-centre cohort of older ASA physical status classification 3–4 patients undergoing major noncardiac surgery, we initially observed a positive association between longer cumulative duration of MAP <65 mm Hg and postoperative delirium after adjusting for patient characteristics and perioperative factors. However, after adjusting for year of surgery, this association was attenuated and no longer statistically significant. Both hypotension exposure and delirium incidence showed a clear downward trend across the study period (Supplementary material 3), suggesting that secular improvements in perioperative blood pressure management, aesthetic techniques, and/or delirium prevention practices may have simultaneously reduced IOH and delirium risk. These findings underscore the importance of accounting for temporal trends when examining associations in retrospective datasets spanning multiple years. Failing to adjust for year can overestimate the contribution of a single exposure, such as IOH, to complex postoperative outcomes such as delirium. Our results suggest that IOH may be more reflective of overall quality of perioperative hemodynamic management in earlier years rather than a direct, independent cause of delirium. Preventing prolonged or profound hypotension remains prudent, but our results indicate that additional reductions in IOH exposure beyond contemporary practice may yield only modest incremental benefit for delirium prevention.

Only few published studies have investigated the association between IOH and delirium in noncardiac surgery, with equivocal findings.^8^, ^9^, ^10^^,^^14^, ^15^, ^16^ These studies considered all patients over a chosen age regardless of their level of health, although one of these studies performed a subgroup analysis on patients with ASA physical classification of 3 or higher. Our results are in accordance with a 2024 study of a cohort of 2352 noncardiac surgical patients older than 70 yr which reported no association between IOH and postoperative delirium, including in a subgroup analysis of patients with ASA scores of 3 or higher.^15^ In contrast, a 2023 report of 605 patients older than 60 yr having thoracic and orthopaedic surgery concluded that IOH >5 min was associated with an increased risk of postoperative delirium.^9^ A third study reported that a MAP <55 mm Hg was associated with a duration-dependent increased risk in postoperative delirium.^10^ The fourth study reported that it was intraoperative blood pressure fluctuation rather than absolute or relative hypotension that was associated with postoperative delirium.^8^ Two recent studies just reported no association between IOH and postoperative delirium.^14^ Our study builds on this existing literature by analysing a large cohort of high-risk ASA physical status 3 or 4 patients 60 yr or older. We report the following variables are linked to increased delirium risk increasing age, male gender, higher ASA physical status, chronic kidney disease, preoperative anaemia and the type of surgery are associated with increased odds of postoperative delirium, which is consistent with the existing body of knowledge on this subject.^23^, ^24^, ^25^, ^26^

Our study included a total of 5171 high-risk patients classified as ASA 3–4 physical status undergoing major noncardiac surgery. We observed an overall postoperative delirium incidence of 11.8%, which is consistent with rates reported in previous studies.^9^^,^^15^^,^^17^^,^^27^

The main strength of this study is its focus on high-risk patients aged 60 and older, providing a more standardised population compared with other studies, and the very low missing data (Table 3). However, there are some limitations to consider. First, as a single-centre retrospective analysis, causal inference cannot be definitively established. Second, although adjusting for year of surgery likely accounts for secular improvements in perioperative blood pressure management, it may also partially over-adjust by removing variance shared between hypotension exposure and delirium incidence. In other words, year of surgery may act as a proxy for multiple unmeasured factors (including anaesthesia practice patterns, hemodynamic targets, monitoring technologies, and postoperative care) that simultaneously influence both IOH exposure and delirium risk. This could attenuate a true physiological association between hypotension and delirium. Nonetheless, given the clear downward trends in both IOH and delirium over time, we believe adjusting for year provides a more conservative and realistic estimate of the independent contribution of IOH. Third, residual confounding by unmeasured variables such as depth of anaesthesia, sedative/anticholinergic exposure, or postoperative complications remains possible. Fourth, limiting inclusion to patients with documented CAM screening may introduce selection bias and restrict generalisability. In addition, although CAM was administered by trained nurses using a standardised tool, we did not formally assess interrater agreement between assessors. Nevertheless, the use of an institutional protocol and embedded EHR templates likely minimised variability. Fifth, we excluded patients with preexisting dementia, cognitive impairment, or previous stroke-TIA. Although this allowed us to better isolate the association between IOH and postoperative delirium, it limits generalisability to the broader population of older adults, in whom such conditions are common. However, this has always been the case in previous published trials on this topic. Future studies are, however, warranted to assess whether IOH poses an even greater risk among cognitively vulnerable individuals. Moreover, inclusion required a documented CAM evaluation; a complete denominator of unscreened eligible patients and their baseline covariates was not available, precluding direct comparison of screened *vs* unscreened populations. This may limit generalisability and introduces the possibility of selection bias related to delirium screening practices. As such, our findings are most applicable to older high-risk adults who underwent routine postoperative delirium screening at our institution. Finally, the single-centre design may limit external validity, and prospective multicentre studies will be necessary to confirm these findings.

### Conclusions

Although intraoperative hypotension initially appeared to be associated with postoperative delirium, this association was no longer significant when accounting for temporal improvements in perioperative care. Intraoperative hypotension may represent a marker of historical practice patterns rather than an independent causal driver of delirium.

## Authors’ contributions

Study design/conception: all authors

Data extraction: TW, IE

Data analysis: AJ, TG

Writing paper: DP, IE, AJ

Editing the paper: all authors

Revising the paper: all authors

## Funding

National Institutes of Health (grant T32GM148369 to NMB).

## Declarations of interest

AJ and MC are consultants for Edwards Lifesciences, Irvine, CA, USA. The other authors declare that they have no conflicts of interest.
