# Supplementary material for: Intraoperative hypotension and postoperative delirium among older high-risk patients undergoing major noncardiac surgery: a retrospective single-centre cohort study
Source: BJA Open. 2025 Oct 15;16:100500. doi: 10.1016/j.bjao.2025.100500 (PMC12550159; doi:10.1016/j.bjao.2025.100500)
Supplement: Multimedia component 2 [file mmc2.docx]

**Supplemental material 2. Covariate definitions used**

| **Covariate** | **Primary definition** | **Supplemental criteria** | **ICD-10 code families / proxies** |
| --- | --- | --- | --- |
| Preoperative midazolam | Any administration of midazolam within 120 minutes prior to anesthesia induction | Dose and route (oral, IV) documented in pre-op MAR | N/A |
| Use of an arterial line | Placement of invasive arterial catheter during case | CPT codes or intraoperative flowsheet entry | N/A |
| Duration of anesthesia | Time from anesthesia start to anesthesia end (minutes) | Extracted from anesthesia record | N/A |
| Age (years) | Age at surgery date (years) | Calculated from DOB | N/A |
| Body mass index | Weight (kg) / [Height (m)]² | Most recent pre-op weight/height within 90 days | N/A |
| ASA physical status | ASA PS score documented preoperatively | If missing, abstracted from anesthesia record | N/A |
| Coronary artery disease | Clinician-documented history of CAD or prior myocardial infarction | Evidence of prior coronary revascularization (PCI, CABG), or positive stress test/angiography showing ≥50% stenosis | I20–I25 |
| Anaemia | Hb < 12.0 (women) or < 13.0 g/dL (men) within 30 days preop | Clinician-documented anemia or ICD-10 code | D50–D64 |
| Arterial hypertension | Clinician-documented HTN or antihypertensive medication use | Two or more pre-op BP ≥ 140/90 mmHg | I10–I15 |
| Diabetes (any types) | Clinician-documented diabetes diagnosis | Hemoglobin A1c ≥ 6.5% within 6 months pre-op | E08–E13 |
| Alcohol Use | Documented use >14 drinks/week (men) or >7 drinks/week (women) | Positive AUDIT-C score | F10.* |
| Drug abuse | Documented illicit drug use or dependence | Positive tox screen (cocaine, amphetamines, opioids) | F11–F16, F18–F19 |
| Depression | Clinician-documented major depressive disorder | Antidepressant medication use within 6 months | F32–F33 |
| Atrial fibrillation (AF) | Clinician-documented AF/flutter | ECG evidence of AF/flutter within 12 months | I48 |
| Use of intraoperative opioids | Administration of any opioid intraoperatively | Dose converted to IV morphine equivalents (mg) | N/A |
| Total fluid in (ml) | Total intraoperative crystalloid, colloid, blood product volume administered | Extracted from anesthesia flowsheet | N/A |
| Blood loss (ml) | Estimated blood loss documented in anesthesia record | If missing, use surgeon-reported EBL | N/A |
| Fluid balance (ml) | Total fluid in − total fluid out (urine + blood loss) | Net balance calculated per case | N/A |
| Total phenyephrine (mcg) | Total cumulative phenylephrine dose intraoperatively | Convert infusion rate to total dose (µg) | N/A |
| Total norepinephrine (mg) | Total cumulative norepinephrine dose intraoperatively | Convert infusion rate to total dose (mg) | N/A |
| Use of intraoperative ketamine | Any administration of ketamine intraoperatively | Dose (mg) and route documented | N/A |
| Year of surgery | Calendar year of procedure | Extracted from anesthesia record | N/A |

*ICD-10 codes refer to the* International Classification of Diseases, 10th Revision *diagnostic codes. Where possible, comorbidities were identified using clinician documentation or laboratory data; ICD-10 code families were used as structured proxies when direct documentation was unavailable to enhance reproducibility*
