# Supplementary material for: Intraoperative hypotension and postoperative delirium among older high-risk patients undergoing major noncardiac surgery: a retrospective single-centre cohort study
Source: BJA Open. 2025 Oct 15;16:100500. doi: 10.1016/j.bjao.2025.100500 (PMC12550159; doi:10.1016/j.bjao.2025.100500)

**Supplementary Material 3** showing delirium and hypotension going down over time

| **Year** | **Mean MAP<65 min (per 60)** | **Delirium %** | **N** |
| --- | --- | --- | --- |
| 2013 | 1.0018 | 20.5 | 73 |
| 2014 | 0.938 | 13.7 | 475 |
| 2015 | 1.1101 | 12.3 | 456 |
| 2016 | 1.0764 | 14.3 | 426 |
| 2017 | 0.8667 | 13.6 | 425 |
| 2018 | 0.7932 | 13.8 | 477 |
| 2019 | 0.7162 | 13.6 | 509 |
| 2020 | 0.6941 | 10.2 | 422 |
| 2021 | 0.6374 | 9.4 | 435 |
| 2022 | 0.5506 | 9.8 | 551 |
| 2023 | 0.5637 | 8.8 | 554 |
| 2024 | 0.5405 | 9.5 | 368 |


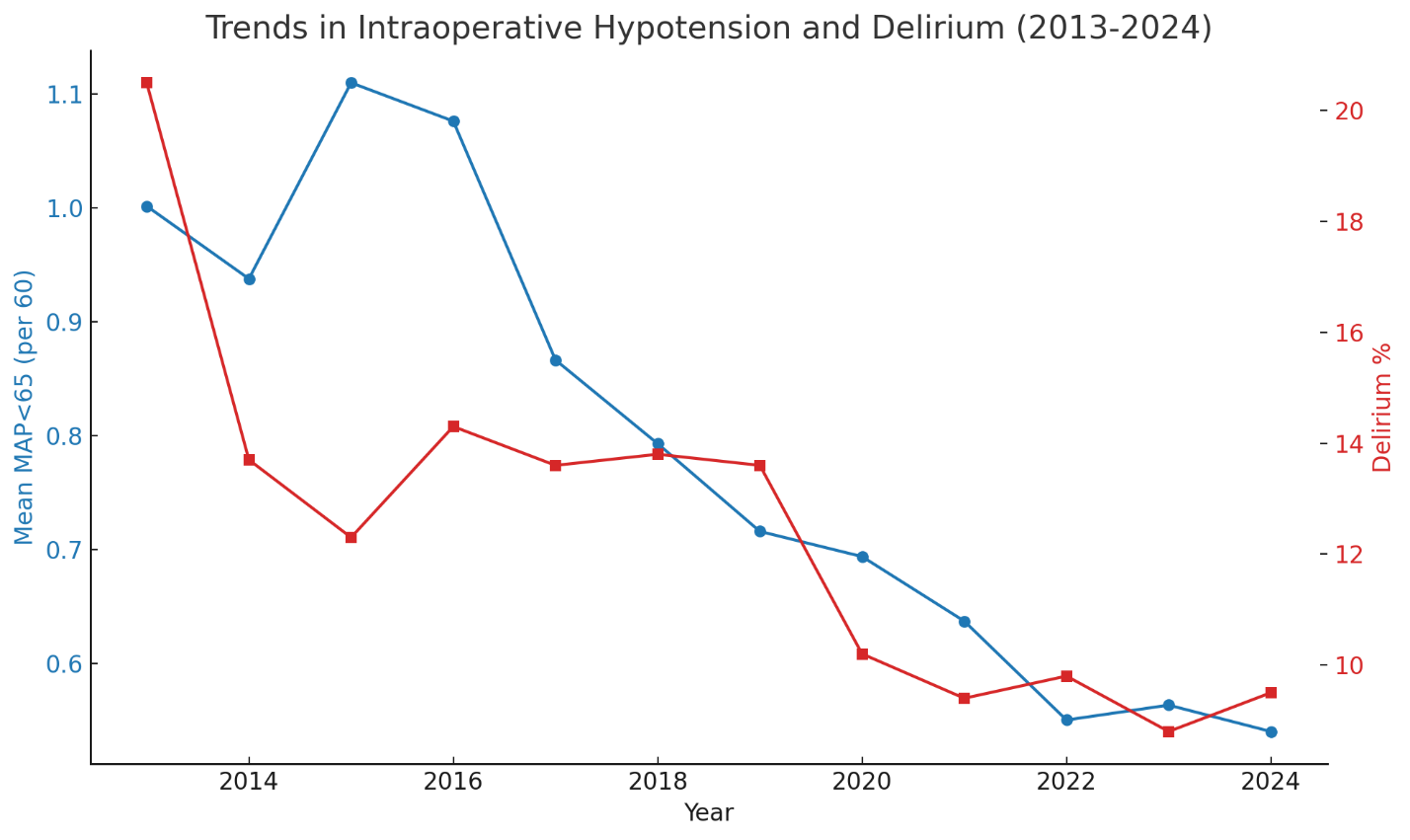

Supplement: Multimedia component 3 [file mmc3.docx]
